# Gehirn-Check-up: strukturiertes Vorgehen zur Diagnostik von kognitiven Störungen im hausärztlichen Setting

**DOI:** 10.1007/s00391-024-02319-y

**Published:** 2024-06-05

**Authors:** Lucas Wolski, Ann-Kathrin Bopp, Ann-Kathrin Schwientek, Sandra Langer, Vildan Dogan, Timo Grimmer

**Affiliations:** 1 Klinik für Psychiatrie und Psychotherapie, Zentrum für kognitive Störungen, Klinikum rechts der Isar, Technische Universität München, Möhlstraße 26, 81675 München, Deutschland; 2 Roche Pharma AG, Grenzach-Wyhlen, Deutschland

**Keywords:** Leichte kognitive Störung, Alzheimer-Demenz, Patientenidentifikation, Primärversorgung, Diagnostischer Algorithmus, Mild cognitive impairment, Alzheimer’s dementia, Identification of patients, Primary care, Diagnostic algorithm

## Abstract

**Hintergrund:**

Die anlassbezogene Identifizierung der leichten kognitiven Störung („mild cognitive impairment“, MCI) in der Primärversorgung ist wichtig, um reversible Ursachen zu behandeln oder das Fortschreiten zu einem demenziellen Syndrom zu verlangsamen. Hierzu wurde die Praxistauglichkeit eines Diagnosealgorithmus, im Weiteren als „Gehirn-Check-up“ bezeichnet, untersucht.

**Methodik:**

Mittels eines standardisierten Fragebogens wurden das Nutzungsverhalten und die Praxistauglichkeit des Gehirn-Check-up in *n* = 37 allgemeinmedizinischen und internistischen Arztpraxen in Deutschland erhoben.

**Ergebnisse:**

Der Gehirn-Check-up wurde von *n* = 37 Ärzt:innen bei 389 Personen (66 %) durchgeführt. Zu den Barrieren bezüglich der Durchführung zählten: Angst der Betroffenen vor dem Ergebnis, Zeitmangel des Fachpersonals sowie Kosten. Insgesamt bewerteten 73 % der Teilnehmenden den Gehirn-Check-up im Behandlungsalltag als praxistauglich. Lange Wartezeiten auf einen Termin bei neurologischen/psychiatrischen Fachärzt:innen wurden als Hürde für eine optimale Betreuung genannt.

**Schlussfolgerung:**

Der strukturierte Algorithmus Gehirn-Check-up scheint hilfreich in der Primärversorgung zu sein, da dieser unter Routinebedingungen im hausärztlichen Setting praxistauglich ist und dazu beitragen kann, anlassbezogen Patient:innen mit einer MCI leichter zu identifizieren. Um weitere Barrieren besser adressieren zu können, bedarf es jedoch weiterer Machbarkeitsstudien.

**Zusatzmaterial online:**

Zusätzliche Informationen sind in der Online-Version dieses Artikels (10.1007/s00391-024-02319-y) enthalten.

## Abstract (en)

**Background:**

The reason-related identification of mild cognitive impairment (MCI) in primary care is helpful to treat reversible causes or decelerate progression to dementia by optimal management of existing risk factors. In this process general practitioners are in a key position. The present feasibility study investigated the practicability of a diagnostic algorithm (brain check-up), comprising neuropsychological examinations, differential diagnoses and follow-up measures.

**Method:**

By means of a standardized questionnaire, the utilization and practicability of the brain check-up was surveyed in *n* = 37 medical practices of general practitioners and internists in Germany.

**Results:**

The brain check-up was performed by *n* = 37 physicians in 389 patients (66%). The main barriers to implementation included patients’ fear of facing the results, the professionals’ lack of time, and costs. Overall, 73% of the participants agreed that the brain check-up was practical in everyday treatment. Long waiting times for an appointment with a neurological/psychiatric specialist were perceived as a barrier for optimal care.

**Conclusion:**

The structured algorithm is convenient in physician’s everyday practice and can contribute to identify patients with MCI more easily. Therefore, it appears to be a helpful tool in primary care. To achieve sustainability in everyday use, identified barriers need to be addressed during the implementation phase.

## Hintergrund und Fragestellung

Nach wie vor sind Frühstadien (leichte kognitive Störungen [„mild cognitive impairment“, MCI]) aus dem Demenzspektrum bei Menschen im höheren Lebensalter unterdiagnostiziert [4, 9, 22]. Folgen, die sich im Laufe der Erkrankung ergeben (psychische und physische Veränderungen), sind nicht nur für die Betroffenen, sondern auch für deren Angehörige belastend [21]. Als erste Ansprechpartner:innen für Betroffene stellen hausärztliche Praxen eine Schlüsselposition dar. Gegenwärtig gibt es jedoch kein einheitliches Vorgehen [29]. Ziel dieses Beitrags ist es, erste Ergebnisse bezüglich der Anwendung eines strukturierten Algorithmus (Gehirn-Check-up) zur anlassbezogenen Identifikation von Patient:innen mit einer MCI und leichtgradig demenziellem Syndrom vorzustellen. Der Algorithmus soll zur Erhöhung des Bewusstseins für die Bedeutung einer zeitgerechten Diagnosestellung im Sinne einer gelingenden Versorgung beitragen. Daneben soll er die Zusammenarbeit der haus- und fachärztlichen Praxen effektiver strukturieren [29].

## Einleitung

### Diagnose MCI – eine Gelegenheit für Interventionen

Mild cognitive impairment bezeichnet die Zwischenphase einer altersbedingten kognitiven Verschlechterung in einem oder mehreren kognitiven Bereichen und einer Demenz [1]. In Abgrenzung zur „subjective cognitive impairment“ (SCI) ist MCI als subjektiv wahrgenommene und in standardisierten Tests objektivierbare kognitive Einbuße bei erhaltener Alltagskompetenz definiert [7]. Sie stellt eines der häufigsten Syndrome bei älteren Menschen dar und geht mit einem erhöhten Demenzrisiko einher [1]. So entwickeln 30–50 % der Patient:innen mit einer MCI innerhalb von 5 bis 10 Jahren und bis zu 10 % bereits innerhalb eines Jahres eine Demenz [17, 30]. MCI muss jedoch nicht immer der Vorläufer einer Alzheimer-Demenz sein. Häufig können auch andere, reversible Ursachen (z. B. Depression, Schilddrüsenunterfunktion etc.) für das klinische Syndrom verantwortlich sein. Folglich ist es notwendig, dass Behandelnde bei Verdacht auf eine kognitive Störung zeitnah einen strukturierten diagnostischen Algorithmus anwenden, um Menschen mit einer MCI individuelle, prospektive Handlungsmöglichkeiten an die Hand zu geben. Denn v. a. im Frühstadium bestehen die günstigsten Chancen, auf die im Krankheitsverlauf auftretenden neuropsychiatrischen Symptome Einfluss zu nehmen [29]. Dies gewährleistet auch, sich verändernde Verhaltensweisen besser einordnen und angemessener (re-)agieren (z. B. Verhaltensinterventionen) zu können [5, 8, 31]. Auch Lifestyle-Veränderungen wie z. B. das Pflegen sozialer Kontakte, Rauchentwöhnung, körperliche und geistige Aktivität, können das Risiko für eine Progression zur Demenz senken [14, 26].

### Barrieren einer MCI-Diagnose im hausärztlichen Setting

Ergebnisse einer qualitativ durchgeführten Studie unter Hausärzt:innen verdeutlichen, dass die Behandlungspfade sowie die Differenzialdiagnostik bei MCI eine Herausforderung darstellen [29]. Werden subjektiv wahrgenommene kognitive Defizite nicht explizit von Betroffenen selbst oder deren Angehörigen erwähnt, stehen sie selten im ärztlichen Fokus. Viele Menschen sind über Risiko- und Protektivfaktoren gering informiert [32]. Ferner bestehen Vorbehalte und Ängste gegenüber einer Untersuchung auf MCI [15, 16]. In der Annahme, dass es keine Möglichkeiten einer Heilung gibt, stellen viele Hausärzt:innen die Sinnhaftigkeit einer Diagnose infrage. Zudem ist die Durchführung kognitiver Tests zeitaufwendig und nicht ausreichend vergütet. Auch erscheinen die Zusammenarbeit und Koordination der Expert:innen untereinander unzureichend [15].

## Methodik

Mit dem Ziel, Betroffene mit einer MCI oder einer Demenz in frühen Erkrankungsphasen zu identifizieren, wurde in Zusammenarbeit mit niedergelassenen neurologisch, psychiatrisch und hausärztlich tätigen Ärzt:innen, der Gehirn-Check-up entwickelt. In der hier beschriebenen Studie werden die Machbarkeit und Akzeptanz des strukturieren Diagnose-Algorithmus untersucht [28]. Näheres zur Methodik kann dem Zusatzmaterial online entnommen werden.

## Ergebnisse

Insgesamt beteiligten sich 37 Ärzt:innen (MW = 55 Jahre) an der Befragung (Tab. 1). Diese betreuten im Quartal 4/2022 insgesamt *n* = 593 Patient:innen mit Verdacht auf eine MCI oder ein demenzielles Syndrom (im Mittel 15,6 Patient:innen/Praxis).

**Tab. 1:** Charakteristika der Stichprobe

Parameter	*n* = 38
*Regionale Verteilung (%)*	
Westliche Bundesländer	37
Südliche Bundesländer	26
Östliche Bundesländer	21
Nördliche Bundesländer	16
*Verteilung, Einwohnerzahl, Stadt/Land (%)*	
< 20.000	26
20.000 bis < 50.000	21
50.000 bis < 200.000	11
200.000 bis < 500.000	11
500.000 bis < 1.000.000	11
> 1.000.000	21

**Abb. 1:**
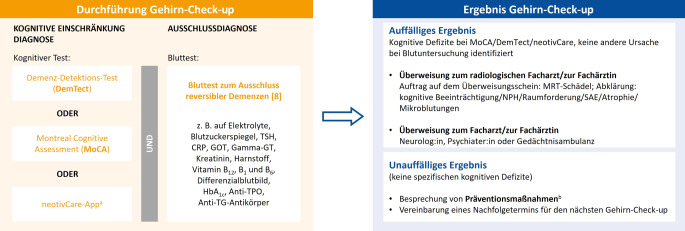
Ablauf des Gehirn-Check-up und Folgemaßnahmen. *CRP* C-reaktives Protein; *GOT* Glutamat-Oxalacetat-Transaminase (Aspartat-Aminotransferase); GT Glutamyltransferase; MRT Magnetresonanztomographie; NPH Normaldruckhydrozephalus; *SAE* subkortikale arteriosklerotische Enzephalopathie; *TG* Thyreoglobulin; *TPO* Thyreoperoxidase; *TSH* thyreoidestimulierendes Hormon. ^a^Geeignet für Patient:innen ab 60 Jahren mit ersten Anzeichen einer leichten kognitiven Einschränkung. ^b^Verbesserung des Gesundheitsverhaltens im mittleren Alter: z. B. körperliche und geistige Aktivität, mediterrane Diät, Reduktion von chronischem Stress, nicht rauchen, Normalgewicht, Hörfunktion erhalten [14].

**Abb. 2:**
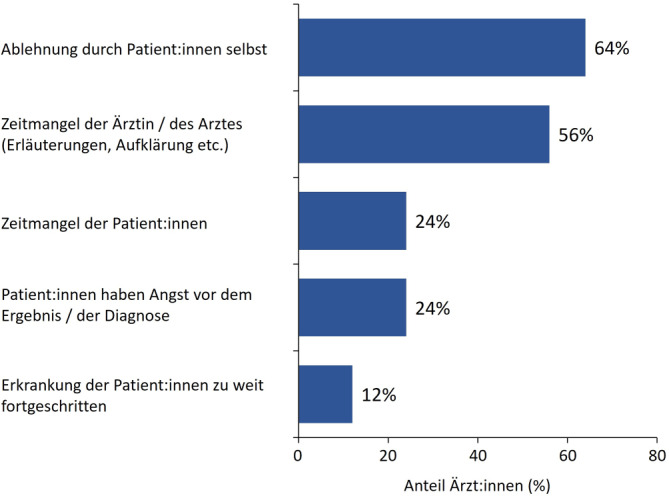
Gründe, warum der Gehirn-Check-up nicht durchgeführt wurde (Mehrfachnennungen möglich, *n* = 25 Ärzt:innen, die nicht bei allen Patient:innen den Gehirn-Check-up durchführten)

**Abb. 3:**
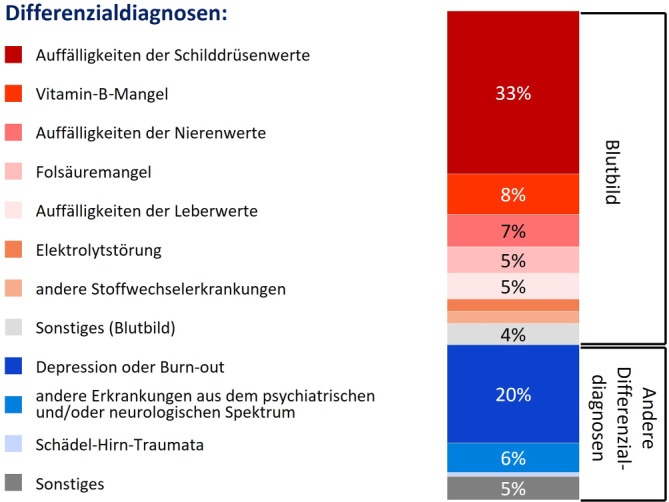
Differenzialdiagnosen als mögliche Ursachen für kognitive Defizite (*n* = 21 Patient:innen mit anderer Ursache für die kognitiven Defizite)

Den Gehirn-Check-up (Abb. 1) führten *n* = 37 Ärzt:innen bei 389 Patient:innen (66 %) durch. Als Gründe, warum 25 der Teilnehmenden den Gehirn-Check-up bei einigen Patient:innen mit subjektiv kognitiven Beschwerden nicht durchführten, wurden in erster Linie Ablehnung durch die betroffenen Personen selbst (64 %) und Zeitmangel der Ärzt:innen (56 %) genannt. Von den Befragten gaben 24 % als Grund an, dass die Betroffenen Angst vor der Diagnose haben (Abb. 2). Insgesamt setzten 95 % der Ärzt:innen den DemTect (Demenz-Detection) [11] und 68 % den MoCA (Montreal Cognitive Assessment) [18] ein. Der DemTect wurde bei 66 %, der MoCA bei 47 % der Patient:innen angewendet. Die Durchführung des DemTect bzw. MoCA erfolgte bei 46 % bzw. 44 % der Befragten durch den:die Ärzt:in und bei 54 % bzw. 56 % durch das Praxispersonal. Zur Nutzung der neotivCare-App (neotiv GmbH, Magdeburg, Deutschland) [2] wurde 18 % (71/389) der Patient:innen von 54 % (20/37) der Ärzt:innen geraten. Ein Bluttest zum Ausschluss von reversiblen Ursachen einer MCI oder eines demenziellen Syndroms wurde bei 77 % der Patient:innen durchgeführt. Mehr als die Hälfte (57 %) der Praxen hatten Patient:innen, bei denen andere mögliche Gründe für die kognitiven Defizite aufgedeckt wurden. Am häufigsten wurden dabei Auffälligkeiten der Schilddrüsenwerte (33 %), Depression oder Burn-out (20 %) und Vitamin-B-Mangel (8 %) identifiziert (Abb. 3).

### Weiterführende Maßnahmen im Rahmen des Gehirn-Check-up

Auf Basis des Gehirn-Check-up hatten 65 % (253/389) der Patient:innen einen auffälligen Befund. Davon wurden 51 % zum:zur neurologischen/psychiatrischen Fachärzt:in oder in die Gedächtnisambulanz, und 40 % zum:zur radiologischen Fach:ärztin überwiesen. Als Gründe gegen eine Überweisung wurden u. a. Ablehnung durch die Betroffenen und Probleme, einen zeitnahen Termin zu erhalten, angegeben. Auch Patient:innen mit unauffälligem Befund (35 %, 136/389) wurden zur Abklärung der Differenzialdiagnostik zum:zur Fachärzt:in überwiesen (davon 23 % in neurologische oder psychiatrische Praxen oder in die Gedächtnisambulanz; 18 % zur radiologischen Untersuchung). Zudem wurden 56 % (142/253) der Patient:innen mit auffälligem und 42 % (57/136) der Patient:innen mit unauffälligem Befund über Präventionsmöglichkeiten aufgeklärt.

### Beurteilung der Praxistauglichkeit

**Abb. 4:**
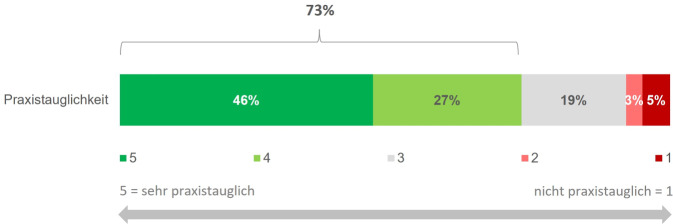
Bewertung der Praxistauglichkeit des Gehirn-Check-up. 73 % (27/37) der Ärzt:innen bewerteten den Algorithmus als praxistauglich

Die Durchführung des DemTect, des MoCA und des Bluttests wurden jeweils von 83 % (29/35), 76 % (19/25) und 90 % (28/31) der Durchführenden als praxistauglich bewertet. Als Herausforderung bei der Durchführung der kognitiven Tests wurde u. a. der Zeitaufwand genannt. Optimierungsmöglichkeiten sahen die Befragten in einer besseren Vergütung. Die neotivCare-App bewerteten 40 % (8/20) der Ärzt:innen, die die App ihren Patient:innen empfohlen haben, als praxistauglich. Zu den Herausforderungen für die Patient:innen zählten laut Ärzt:innen fehlende Smartphones, wenig Erfahrung mit Apps sowie zu komplizierte Handhabung und Aufgaben. Beim ärztlichen Personal sprach hauptsächlich der Zeitaufwand für Erläuterungen gegen den Einsatz der App. Insgesamt beurteilten 73 % der Ärzt:innen den Gehirn-Check-up als praxistauglich (Abb. 4). Als häufigste Hürden für dessen Durchführung wurden die Wartezeiten bei dem:der Fachärzt:in, die Ablehnung der Untersuchung aus Angst vor dem Ergebnis und den möglichen Konsequenzen sowie die Kosten der Durchführung identifiziert.

## Diskussion

Insgesamt wurde der Gehirn-Check-up durch die teilnehmenden Ärzt:innen als praxistauglich bewertet. Am häufigsten wurde der DemTect, gefolgt vom MoCA, (bei 66 % bzw. 47 % der Patient:innen) eingesetzt. Die App neotivCare spielte mit einem Anteil unter 20 % eine eher untergeordnete Rolle. Dies kann sich zum einen auf die fehlende technische Kompetenz der Betroffenen als auch auf Schwierigkeiten im Umgang mit der App zurückführen lassen [13, 19]. Obwohl die App im häuslichen Umfeld durch Patient:innen selbst angewendet wird, sahen die Ärzt:innen einen hohen Zeitaufwand für Erläuterungen als kritisch an.

Als Barrieren gegen die Durchführung des entwickelten Gehirn-Check-up wurden der Zeitaufwand für die Ärzt:innen und die nichtausreichende Vergütung identifiziert. Hinsichtlich zeitökologischer Aspekte verweisen die NICE(National Institute for Health and Care Excellence)-Leitlinien (2018) auf verschiedene neuropsychologische Kurztests, die lediglich 3 min umfassen, wie z. B. den Mini-Cog [20]. Dies könnte zu einer höheren Akzeptanz beitragen, allerdings umfassen diese nicht alle kognitiven Bereiche die, bei einer frühen Diagnostik, besonders relevant sind [7]. Eine weitere Barriere ist die Ablehnung durch die Betroffenen, da diese Angst vor dem Ergebnis und den möglichen Konsequenzen haben. Diese Erkenntnis stimmt mit publizierten Aussagen zur Einstellung von Patient:innen und Behandelnden bezüglich einer Demenzdiagnostik überein [15, 16]. Bei einem auffälligen Befund durch den Gehirn-Check-up wurde nur die Hälfte der Patient:innen an neurologische/psychiatrische Praxen bzw. in eine Gedächtnisambulanz überwiesen, häufig aufgrund von Ablehnung der Betroffenen oder aufgrund von Problemen bei der Terminfindung. Hier besteht Optimierungsbedarf durch eine bessere Interaktion zwischen Haus- und Fachärzt:innen. Diese Beobachtung deckt sich mit Ergebnissen einer kürzlich durchgeführten Umfrage der Autor:innen unter niedergelassenen Haus- und Fachärzt:innen zu Herausforderungen und Barrieren in der Diagnostik von MCI und leichten demenziellen Syndromen (unveröffentlichte eigene Daten).

### Differenzialdiagnostik hat hohe Relevanz

Da eine kognitive Einschränkung auch behandelbare Ursachen haben kann [14], ist eine Differenzialdiagnostik wichtig. Ein großer Teil reversibler Ursachen kann mittels Bluttests identifiziert werden (z. B. Schilddrüsenwerte oder Vitamin‑B_12_-Mangel). In der vorliegenden Befragung wurde ein Bluttest bei 77 % der Patient:innen durchgeführt. Ebenso kann eine Überweisung an Fachärzt:innen bei einem auffälligen Befund sinnvoll sein, um Erkrankungen aus dem psychiatrisch/neurologischen Spektrum abzuklären. Nur etwa mit 50 % der Betroffenen wurden Präventionsmaßnahmen besprochen. Hier besteht sowohl für Arztpraxen als auch für Patient:innen Aufklärungsbedarf darüber, dass MCI und das Voranschreiten zu einer Demenz durch die Modifizierung von Risikofaktoren beeinflusst werden können [14, 25, 26, 32]. Die Alzheimer-Krankheit als häufigste Ursache einer MCI ist derzeit nicht heilbar. Neben den Acetylcholinesterasehemmern, die den Krankheitsverlauf bei leichten bis mittelgradigen Einschränkungen wirksam verlangsamen, befindet sich eine Reihe von Wirkstoffen in der klinischen Entwicklung. Deren Ziel ist es, den Verlauf der Erkrankung in einem frühen Stadium zu verlangsamen und damit die Entwicklung eines demenziellen Syndroms zu verzögern [6].

### Die Schlüsselrolle von Hausärzt:innen bei der Diagnostik einer MCI

Hausärzt:innen nehmen eine besondere Rolle bei der Diagnostik ein, da sie eine erste Anlaufstelle für Betroffene und deren Angehörige bieten [10]. Mit einem strukturierten diagnostischen Algorithmus könnten Betroffene besser identifiziert und zur weiteren Abklärung an Spezialist:innen überwiesen werden. Eine rechtzeitige Diagnose ermöglicht zudem, Lebensstilveränderungen vorzunehmen und die Zukunft zu planen [23]. Da einer nationalen Studie [31] zufolge derzeit die mittlere Zeit vom Auftreten der ersten Symptome bis zur korrekten Diagnose 16 Monate beträgt, ist eine Optimierung der Patientenpfade dringend erforderlich. Screeningprogramme auf Demenz sind bereits in vielen Ländern mit hohem Einkommen Bestandteil der jährlichen geriatrischen Versorgung [12]. Awareness-Kampagnen in Analogie zu den gängigen Vorsorgeuntersuchungen könnten dazu beitragen, die Bevölkerung für Maßnahmen bei kognitiven Defiziten zu sensibilisieren. Ein breites Bevölkerungsscreening asymptomatischer Betroffener > 65 Jahre ist jedoch aufgrund fehlender Evidenz eines Benefits gegenüber einem potenziellen Schaden umstritten [24]. Anstatt eines generellen kognitiven Screenings wird empfohlen, Personen zu untersuchen, die selbst oder deren Hausärzt:innen Bedenken bezüglich ihrer kognitiven Leistung haben [27]. Voraussetzung dafür sind profunde Kenntnisse über das Syndrom MCI und dessen Abgrenzung zur Demenz [3].

### Zusammenfassung

Auch wenn der Diagnosealgorithmus von den Ärzt:innen als insgesamt praxistauglich beurteilt wurde, so müssen dennoch verschiedene zeitliche, organisatorische und finanzielle Barrieren adressiert werden, bevor er Einsatz in der Routineversorgung finden kann. Hierbei muss v. a. das Setting der allgemeinmedizinischen Versorgung Beachtung finden. Für eine nachhaltige Etablierung müssen, unter Einbindung der Stakeholder, finanzielle Anreize sowie Maßnahmen für eine verbesserte Kommunikation der Behandelnden untereinander etabliert werden [28].

### Limitationen der Analyse

Die Anzahl der Patient:innen mit Verdacht auf eine kognitive Störung pro teilnehmender Praxis innerhalb des Testzeitraums wies, je nach Größe der Praxis, eine breite Streuung auf. Dies kann Einfluss auf die prozentuale Verteilung der Antworten haben. Die Erhebung mittels Fragebogen könnte zu eingeschränkten Antworten bzw. undifferenzierten Prüfungen der Ursachen, wie beispielsweise Zeitmangel, geführt haben. Während alle Teilnehmenden die Einführung zur Durchführung des Gehirn-Check-up im Rahmen des „Kick-off“ besuchten, war eine weitere Schulung zur Durchführung der kognitiven Tests sowie zur Anwendung der neotivCare-App freiwillig und wurde nur teilweise besucht. Dies kann Einfluss auf die korrekte Durchführung der kognitiven Tests nehmen.

## Fazit für die Praxis

- Die Ergebnisse deuten darauf hin, dass ein strukturierter Algorithmus (hier: Gehirn-Check-up) zur Anwendung bei symptomatischen Patient:innen auch unter Routinebedingungen im hausärztlichen Alltag praxistauglich sein kann.
- Die Wirksamkeit des Algorithmus im Vergleich zum Vorgehen in der Routineversorgung in der Identifikation von MCI-Patient:innen muss jedoch in weiteren Studien untersucht werden.
- Die genannten Barrieren (Zeit, Ängste der Betroffenen, mangelnde Kenntnis bezüglich MCI etc.) müssen bei der Implementierung adressiert werden, um eine nachhaltige Anwendung im Praxisalltag zu ermöglichen.

## Supplementary Information

Methodik
