# Supplementary material for: Brain check-up: a structured approach diagnosing mild cognitive impairment in the primary care setting
Source: Z Gerontol Geriatr. 2024 Jun 5;57(8):645–51. [Article in German] doi: 10.1007/s00391-024-02319-y (PMC11602870; doi:10.1007/s00391-024-02319-y)
Supplement: Supplementary file 1 — Methodik [file 391_2024_2319_MOESM1_ESM.docx]

# Methodik

# Ziel und Ablauf des Gehirn-Check-Ups

Bei Betroffenen mit subjektiv geäußertem Verdacht auf kognitive Defizite wurde in einem ersten Schritt, eines von drei kognitiven Diagnoseinstrumenten: Demenzdetektionstest (DemTect), Montreal Cognitive Assessment (MoCA) oder neotivCare App durchgeführt (DIGA). Zweitens erfolgte ein Bluttest zum Ausschluss reversibler Ursachen (vgl. Abbildung 1). Zuletzt wurden die Ergebnisse des Bluttests und des neuropsychologischen Kurztests mit dem/der Patient:in besprochen. Bei DemTect und MoCA handelt es sich um validierte Kurztests mit einer hohen Sensitivität (MoCA: 90%, DemTect: 80%) bzw. Spezifität (MoCA: 87%) [11, 18]. Diese werden bereits in der Routineversorgung für die Identifikation von MCI und dementiellen Syndromen eingesetzt. Die Kurztests werden in der Regel von medizinischem Fachpersonal durchgeführt. Die neotivCare App wurde 2021 entwickelt und zeigt eine Sensitivität von 0,82 und eine Spezifität von 0,71 [2]. Die Durchführung liegt hier bei dem:der Patient:in, der entsprechend den Vorgaben für DIGAs den Befundbrief bekommt, und diesen aktiv bei dem:der behandelnden Ärzt:in anspricht. Bezüglich der Anwendung im Praxisalltag erhielten die Ärzt:innen eine entsprechende Schulung. Zudem wurde eine weitere (nahestehende) Person bezüglich der Funktionalität angeleitet. Nach einer Unterweisung wurde ein Code, für die Nutzung der App, an die Patiennt:innen ausgegeben. Bei Identifizierung kognitiver Defizite - entsprechender Wert in einem neuropsychologischen Kurztest (z.B. MoCA: ≤26) oder einem Hinweis aus der neotivCare App (Angabe eines Composite Scores, der das Testergebnis des:der Patient:in ins Verhältnis zum Normwert der Ergebnisse von kognitiv unbeeinträchtigten Personen der entsprechenden Altersgruppe setzt) und dem Ausschluss anderer möglicher Erkrankungen, erfolgte zur weiteren Abklärung eine Überweisung in die Neurologie, Psychiatrie oder Gedächtnisambulanz sowie eine radiologische Abklärung, u.a. zum Ausschluss von Normaldruckhydrozephalus (NPH), Raumforderungen, subkortikalen arteriosklerotischen Enzephalopathien (SAE), Atrophie und Mikroblutungen. Bei einem unauffälligen Befund sollten Gesundheitsverhalten modifizierende Maßnahmen besprochen und ein Anschlusstermin vereinbart werden.

# Datenerhebung, -analyse und Stichprobenbeschreibung

Die Datenerhebung erfolgte in einem dreistufigen interaktiven Vorgehen mit n=37 Hausärzt:innen und hausärztlich tätigen Internist:innen in Deutschland in einem Zeitraum von August bis Dezember 2022. In jeder der drei Testphasen wendeten teilnehmende Ärzt:innen den diagnostischen Algorithmus im Praxisalltag an. Im Anschluss wurde die Durchführbarkeit untersucht und der Algorithmus optimiert. In der aktuellen Arbeit werden die Ergebnisse der dritten abschließenden Testphase berichtet. Eingeschlossen wurden Allgemeinmediziner:innen und hausärztlich tätige Internist:innen mit mindestens drei Jahren Praxiserfahrung. Die Rekrutierung erfolgte Deutschlandweit über alle Altersklassen hinweg über das Marktforschungsinstitut Produkt + Markt GmbH & Co. KG. Das Institut betreut einen festen Pool an Ärzt:innen, der ca. 15.000 Einträge aus allen Facharztrichtungen umfasst. Die Ärzt:innen, die die Rekrutierungskriterien erfüllten, wurden per Mail und Telefon kontaktiert und bei Interesse die Befragungsgruppe aufgenommen. Vor Beginn der ersten Testphase fand ein für alle Teilnehmenden verpflichtendes 90-minütiges virtuelles Kick-Off-Meeting statt, in dem unter anderem die Studienrationale, der Algorithmus Gehirn-Check-Up und dessen Anwendungsbereich erläutert wurden. Daneben erhielten die Ärzt:innen Schulungen zu einzelnen Komponenten, wie z.B. DemTect [11], MoCA [18] oder der App neotivCare [2]. Mittels eines standardisierten 13-Item-Fragebogens wurde die Anzahl der Patient:innen, bei denen der Gehirn-Check-Up vollständig oder in Teilen durchgeführt wurde sowie Gründe für ein Nicht-Durchführen erhoben. Daneben wurden die von den Ärzt:innen auf Basis der Ergebnisse des Gehirn-Check-Ups abgeleiteten Differenzialdiagnosen und eingeleiteten Folgemaßnahmen erfragt. Die Teilnemenden bewerteten die Praxistauglichkeit sowie Hürden der Durchführung einzelner Prozessschritte auf einer 5-stufigen Likertskala (1=nicht praxistauglich bis 5=sehr praxistauglich; 1=große Hürde bis 5=keine Hürde). Bewertungen der Kategorie 4 und 5 wurden als praxistauglich eingestuft. Die Teilnahme an der quantitativen Befragung erfolgte freiwillig und anonym. Der Fragebogen wurde deskriptiv mit GESStabs (GESS Gesellschaft für Software in der Sozialforschung mbH, Hamburg; Version 5.2.0.12). Für kontinuierliche Variablen wurden statistische Parameter einschließlich Mittelwert, Standardabweichung, Median und Spanne berechnet und Häufigkeitsverteilungen für diskrete Variablen als Prozentsatz im Verhältnis zur Gesamtstichprobe angegeben.
